# Exercise-induced expression of angiogenic growth factors in skeletal muscle and in capillaries of healthy and diabetic mice

**DOI:** 10.1186/1475-2840-7-13

**Published:** 2008-05-01

**Authors:** Riikka Kivelä, Mika Silvennoinen, Maarit Lehti, Sanni Jalava, Veikko Vihko, Heikki Kainulainen

**Affiliations:** 1 LIKES Research Center for Sport and Health Sciences, Rautpohjankatu 8a, 40700 Jyväskylä, Finland; 2 Neuromuscular Research Center, Department of Biology of Physical Activity, P.O. Box 35, 40014 University of Jyväskylä, Finland; 3 Institute of Medical Technology, University of Tampere and Tampere University Hospital, Tampere, Finland

## Abstract

**Background:**

Diabetes has negative, and exercise training positive, effects on the skeletal muscle vasculature, but the mechanisms are not yet fully understood. In the present experiment the effects of running exercise on the mRNA expression of pro- and antiangiogenic factors were studied in healthy and diabetic skeletal muscle. The responses in capillaries and muscle fibers, collected from the muscle with laser capture microdissection, were also studied separately.

**Methods:**

Healthy and streptozotocin-induced diabetic mice were divided into sedentary and exercise groups. Exercise was a single bout of 1 h running on a treadmill. Gastrocnemius muscles were harvested 3 h and 6 h post exercise, and angiogenesis-related gene expressions were analyzed with real-time PCR. In addition to muscle homogenates, capillaries and muscle fibers were collected from the muscle with laser capture microdissection method and analyzed for vascular endothelial growth factor-A (VEGF-A) and thrombospondin-1 (TSP-1) mRNA expression.

**Results:**

Of the proangiogenic factors, VEGF-A and VEGF receptor-2 (VEGFR-2) mRNA expression increased significantly (*P *< 0.05) in healthy skeletal muscle 6 h post exercise. VEGF-B also showed a similar trend (*P *= 0.08). No significant change was observed post exercise in diabetic muscles in the expression of VEGF-A, VEGFR-2 or VEGF-B. The expression of angiogenesis inhibitor TSP-1 and angiogenic extracellular matrix protein Cyr61 were significantly increased in diabetic muscles (*P *< 0.05–0.01). Capillary mRNA expression resembled that in the muscle homogenates, however, the responses were greater in capillaries compared to muscle homogenates and pure muscle fibers.

**Conclusion:**

The present study is the first to report the effects of a single bout of exercise on the expression of pro- and antiangiogenic factors in diabetic skeletal muscle, and it provides novel data about the separate responses in capillaries and muscle fibers to exercise and diabetes. Diabetic mice seem to have lower angiogenic responses to exercise compared to healthy mice, and they show markedly increased expression of angiogenesis inhibitor TSP-1. Furthermore, exercise-induced VEGF-A expression was shown to be greater in capillaries than in muscle fibers.

## Background

Diabetes is a risk factor for peripheral vascular diseases, and it is associated with impaired collateral vessel growth in skeletal muscle [1,2]. Angiogenesis, the growth of new blood vessels from pre-existing ones, is controlled by complex pathways with both pro- and antiangiogenic factors [3]. Recently, both type 1 and type 2 diabetes have been shown to affect angiogenic growth factors and inhibitors in skeletal muscle [4,5]. In our previous study streptozotocin-induced diabetes decreased the mRNA levels of several proangiogenic proteins and increased those of antiangiogenic ones in mouse skeletal muscle [4]. This change in the balance between stimulators and inhibitors may be one of the reasons for the markedly increased risk for peripheral cardiovascular complications in diabetes.

The effects of exercise on angiogenesis in skeletal muscle are known to be opposite to those of diabetes. Exercise-induced increase in skeletal muscle capillarization is a well-known phenomenon in healthy humans and in animals [6], and it has been linked to the increased expression of angiogenic growth factors after exercise. Diabetes, in turn, has been demonstrated to impair skeletal muscle and cardiac angiogenesis, and the mechanisms underlying this have generated much interest recently [2,7-12]. Studies on capillary density and changes in capillarization after endurance exercise training in diabetic animals and patients have produced conflicting results [13-15]. Our earlier results showed exercise training to have some positive effects on the basal levels of angiogenic growth factors in diabetic skeletal muscle [4], and this is to our knowledge the only study to present exercise training-induced changes in the angiogenic factors in diabetic skeletal muscle. The acute effects in diabetic muscle have not been reported. In healthy skeletal muscle several animal and human studies have shown increased VEGF-A expression after acute exercise [16-18] or electrical stimulation [19,20].

In addition to muscle fibers, several other cell types are present in the extracellular matrix, such as endothelial cells, pericytes, and fibroblasts. Skeletal muscle angiogenesis has previously been studied using muscle homogenates, which contain all the cell types present in the muscle tissue. However, in the case of angiogenesis it is of interest to study responses in capillary endothelial cells and in muscle fibers separately. Both these cell types are known to produce vascular endothelial growth factor -A (VEGF-A), which is considered the main angiogenic growth factor. Another approach has been to study endothelial cells and myocytes in the cell culture, but this does not represent the *in vivo *environment in the muscle. Recently, Milkiewicz and Haas demonstrated the feasibility of the laser capture microdissection (LCM) method in the study of capillary-specific gene expression from heterogeneous tissue such as skeletal muscle [21]. They showed that in capillaries, which were isolated from overloaded skeletal muscles, mechanical stretch increased the expression of HIF-1α and MMP-2. This method provides a tool to study responses separately in different cell types from heterogeneous tissues.

Our exercise training study suggested that regular training could increase the basal mRNA levels of angiogenic growth factors in diabetic muscle. In the present study the aim was to compare the responses of pro- and antiangiogenic factors to a single bout of running exercise in healthy and diabetic muscle to exercise. In healthy skeletal muscle the acute proangiogenic responses have been studied, but the responses in diabetic muscles have not been presented. In addition to skeletal muscle homogenates we studied the expression levels separately in capillaries within the muscle and in pure muscle fibers using the LCM method. We focused on the recovery period 3 h and 6 h post exercise, when angiogenesis processes in capillaries are likely to occur.

## Methods

### Experimental set-up

All experimental procedures were approved by the Animal Care and Use Committee of the University of Jyväskylä, Finland. Animals were housed in standard conditions (temperature 22°C, humidity 60 ± 10%, light from 8.00 am to 8.00 pm), and they had free access to tap water and food pellets (R36, Labfor, Stockholm, Sweden).

Adult male NMRI mice (n = 48, Harlan, the Netherlands) aged 10–15 weeks were randomly assigned into healthy and diabetic groups. The diabetic group received a single peritoneal injection of streptozotocin (STZ, Sigma-Aldrich, France, 180 mg/kg) dissolved in sodium citrate buffer solution (0.1 mol/l, pH 4.5) to induce experimental type 1 diabetes. The other group received a sham injection of an equal volume of the buffer. Diabetes was confirmed three days after the injection by a blood glucose test (HemoCue B-Glucose analyzer, Ängleholm, Sweden). Mice were characterized as diabetic when their blood glucose values were greater than 15 mmol/L, and they were not treated with insulin during the experiment. Ten days after injections the mice were divided into three healthy and three diabetic groups (n = 8). One healthy and one diabetic group served as sedentary controls without exercise (H and D), and the four other groups performed 1 h running exercise on a treadmill (speed 21 m/min with an incline of 2.5°). One healthy and one diabetic exercise group were sacrificed 3 h after the end of the exercise (HE3 and DE3), and the other two groups 6 h after (HE6 and DE6). Before the actual experiment all mice were familiarized with treadmill running. Only those mice, which were able to run on a treadmill were chosen and further divided into the different groups. About 10% of the mice were excluded from the experiment, because of their inability to run on a treadmill.

### Tissue preparation

Mice were killed by cervical dislocation and gastrocnemius muscles were immediately removed. The proximal part of the right gastrocnemius muscle was mounted in an O.C.T. embedding medium (Miles, Elkhart, USA) under a microscope to orientate muscle fibers vertically and snap frozen in liquid nitrogen-cooled isopentane. The distal part of the muscle was snap frozen in liquid nitrogen and stored at -80°C for further analysis. The heterogeneity of the muscle was taken into account by taking whole cross-sections for RNA extraction from the same part of the muscle.

### RNA extraction

Total RNA was isolated from the gastrocnemius muscle with Trizol Reagent (Invitrogen, Carlsbad, USA) according to the manufacturer's protocol. The concentration and purity of RNA was determined spectrophotometrically at the wavelengths 260 and 280 nm. Integrity was checked with agarose gel electrophoresis. RNA was reverse-transcribed to cDNA with a High Capacity cDNA Archive Kit (Applied Biosystems, Foster City, USA).

### Laser capture microdissection (LCM)

LCM was used to collect capillaries and pure muscle fibers from the gastrocnemius muscle. Three mice from groups C, D, HE6 and DE6, comprising a total of 24 LCM samples (capillaries and muscle fibers from each animal), were analyzed for the GAPDH-normalized expression of VEGF-A and TSP-1 in capillaries and muscle fibers. The procedures used to collect capillaries and muscle fibers from skeletal muscle were adopted from Milkiewicz & Haas (2005) [21]. Cryosections (8 μm) were cut on uncoated glass slides, immediately fixed with cold acetone and stored at -80°C. Samples were further processed within two days. All equipment was treated with a RNase Away-solution (QBiogene, Morgan Irvine, CA) to prevent RNA degradation. Immediately after taking the samples out of the freezer, capillaries were stained with Isolectin GS-IB_4 _from Griffonia simplicifolia Alexa Fluor 488 conjugate (Molecular Probes), which was diluted in sterile PBS with SUPERaseIn RNAase inhibitor (Ambion, Austin, USA). After a brief wash the samples were properly dehydrated and debris was removed with PrepStrip (Arcturus Engineering, Molecular Devices Corporation, Sunnyvale, USA). Laser capture microdissection for capillaries and pure muscle was performed with a Veritas microdissection system (Arcturus Engineering). From each sample 750–1000 capillaries and 1100–2300 similar laser pulses from the muscle fibers were randomly collected with CapSure LCM caps (Arcturus Engineering) from all parts of the muscle, including both the deep oxidative and the superficial glycolytic portions. Capillaries were distinguished by size (<10 μm) from larger blood vessels. RNA was extracted with a PicoPure RNA isolation kit (Arcturus Engineering) according to the protocol. RNA was transcribed to cDNA with a Sensiscript reverse transcription kit (Qiagen) at 42°C for 2 h, using both random and oligo (dT) primers (Ambion).

### Real-time quantitative PCR

The ABI Prism Sequence Detection System 7300 (Applied Biosystems) was used to perform TaqMan probe-based real-time PCR reactions. TaqMan primer and probe sets for VEGF-A (Mm00437304_m1), VEGF-B (Mm00442102_m1), VEGFR-1 (Mm00438980_m1), VEGFR-2 (Mm00440099_m1), TSP-1 (Mm00449022_m1) and Cyr61 (Mm00487498_m1) were designed and synthesized by Applied Biosystems. Primer pairs were designed so that they overlapped an exon-exon boundary to avoid interference from possible genomic DNA contamination. As an endogenous control to correct for potential variation in RNA loading, GAPDH (Mm99999915_g1) was used as a housekeeping gene. GAPDH was chosen among the commonly used housekeeping genes based on the microarray results from our previous study with similar experimental diabetes and exercise protocol [4], and a study by Yechoor et al. [22] with similar streptozotocin-induced diabetes and skeletal muscle samples. Furthermore, it is considered the most stable internal control in endurance exercise studies [23,24]. The stability of the GAPDH expression was also examined between the experimental groups in relation to the amount of total RNA. ANOVA P-value was 0.988 and the mean values and SD were very similar in all groups, indicating that GAPDH was not affected by diabetes or exercise in our samples. Target genes in the muscle homogenates were quantified according to the corresponding gene-specific standard curve. The expression of VEGF-A and TSP-1 was analyzed in the LCM samples. The comparative C_T _method was utilized for the LCM samples as outlined in the Applied Biosystems User Bulletin 2 (Applied Biosystems). The amplification efficiencies of the target genes and GAPDH were checked to be equal over a range of serial dilutions. All samples were analyzed in triplicate.

### Statistical analysis

Statistical analyses were carried out using SPSS for Windows statistical software release 13.0 (SPSS, Chigago, IL, USA). Data were analyzed for normality and the non-parametric Kruskall-Wallis test with Mann-Whitney U tests were used to analyze differences between groups. Pearson correlation was used to detect similar expression patterns between the studied genes and glucose concentrations. The significance level was set at *P *< 0.05. Changes in the GAPDH-normalized mRNA expressions are expressed as fold-change from healthy control, which was set to 1.

## Results

### Blood glucose and body weight

Whole blood glucose was significantly higher in the diabetic than healthy mice (35.6 ± 6.7 (mean ± SD) vs. 8.1 ± 1.1 mmol/L, *P *< 0.001) on the day of the experiment. The diabetic and healthy mice did not differ in body weight at the beginning of the experiment (36.8 ± 2.3 vs. 36.9 ± 3.2 g), but after ten days of diabetes, the diabetic mice had significantly reduced body weight compared to the healthy mice (33.1 ± 4.1 vs. 37.9 ± 3.3 g, *P *< 0.001).

### Messenger RNA in skeletal muscle homogenates

**Figure 1:**
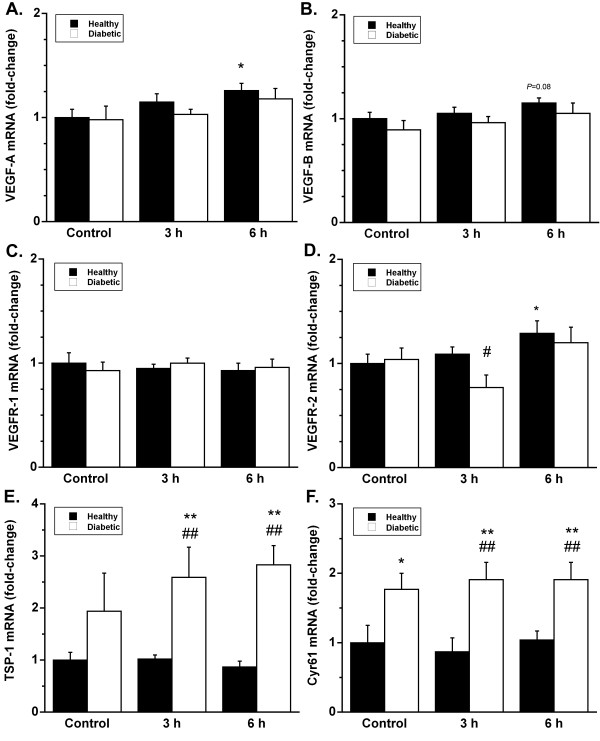
Effects of 1 h running exercise on skeletal muscle mRNA expression of VEGF-A, VEGF-B, VEGFR-1, VEGFR-2, TSP-1 and Cyr61 in healthy (black bars) and diabetic (white bars) mice. The results are expressed as a fold-change (mean ± SE) from the healthy control group, which is set to 1. * *P *< 0.05 vs. healthy control, ** *P *< 0.01 vs. healthy control, # *P *< 0.05 vs. respective healthy exercised group, ## *P *< 0.01 vs. respective healthy exercised group.

VEGF-A and VEGFR-2 mRNA expression increased significantly (*P *< 0.05) in the healthy mice 6 h post exercise (Fig. 1). VEGF-B also showed similar trend at 6 h post exercise, but the increase was not statistically significant (*P *= 0.08). No significant change was observed in the diabetic mice in the expression of VEGF-A, VEGFR-2 or VEGF-B post exercise. VEGFR-1 expression was not affected by exercise in either healthy or diabetic mice.

The expression of TSP-1 was increased in exercised diabetic groups DE3 and DE6 compared to the healthy control group and the respective healthy exercised groups (*P *< 0.01) (Fig. 1). The diabetic sedentary mice (D) also had higher expression compared to the healthy control group, but this was not statistically significant due to the large variation. Diabetes also increased the expression of Cyr61 (Fig. 1). Cyr61 expression was higher in all the diabetic groups compared to the healthy control group and the respective exercise groups (*P *< 0.05–0.01).

**Figure 2:**
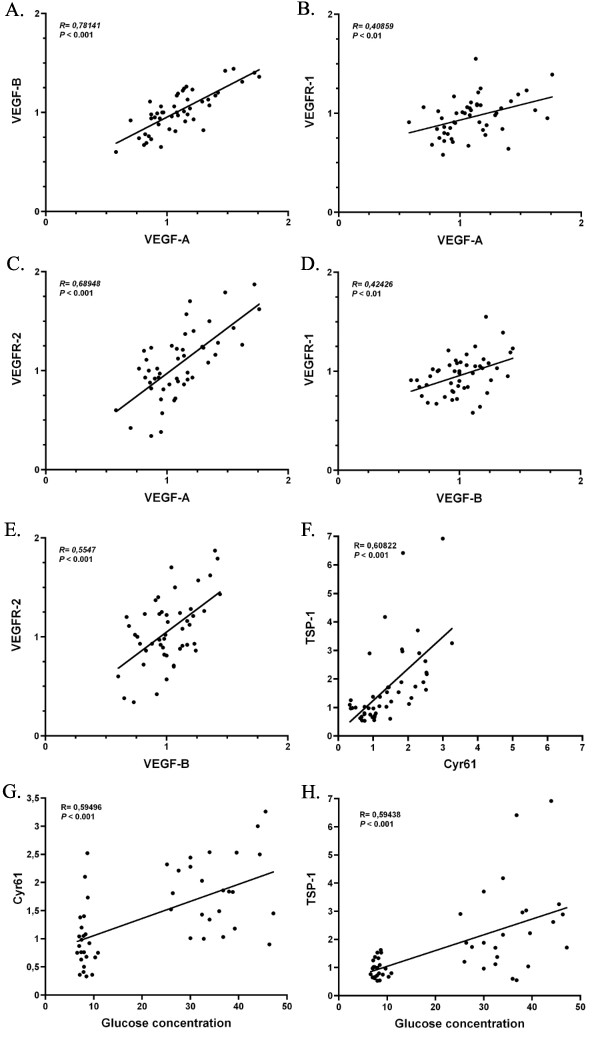
The expression levels of the angiogenic growth factors VEGF-A and VEGF-B and their receptors VEGFR-1 and VEGFR-2 correlated positively across the study population (A-E). TSP-1 and Cyr61 correlated positively with blood glucose concentration and also with each other (F-H). In Cyr61 and TSP-1 the variation between diabetic mice was markedly larger than between healthy mice, as can be seen in images g and h.

VEGF-A expression levels correlated positively with the expression of VEGF-B (*P *< 0.001), VEGFR-1 (*P *< 0.01) and VEGFR-2 (*P *< 0.001) (Fig. 2). VEGF-B also correlated positively with both VEGFR-1 and -2 (*P *< 0.01-0.001). High glucose levels were associated with increased expression levels of TSP-1 and Cyr61 (*P *< 0.001) and decreased expression levels of VEGF-B (*P *< 0.05). TSP-1 and Cyr61 expression also correlated strongly (*P *< 0.001) with each other. The variation in the expression of Cyr61 and TSP-1 was markedly greater in the diabetic than in the healthy mice, as can be seen in figures 2g and 2h.

### Messenger RNA in capillaries and muscle fibers

**Figure 3:**
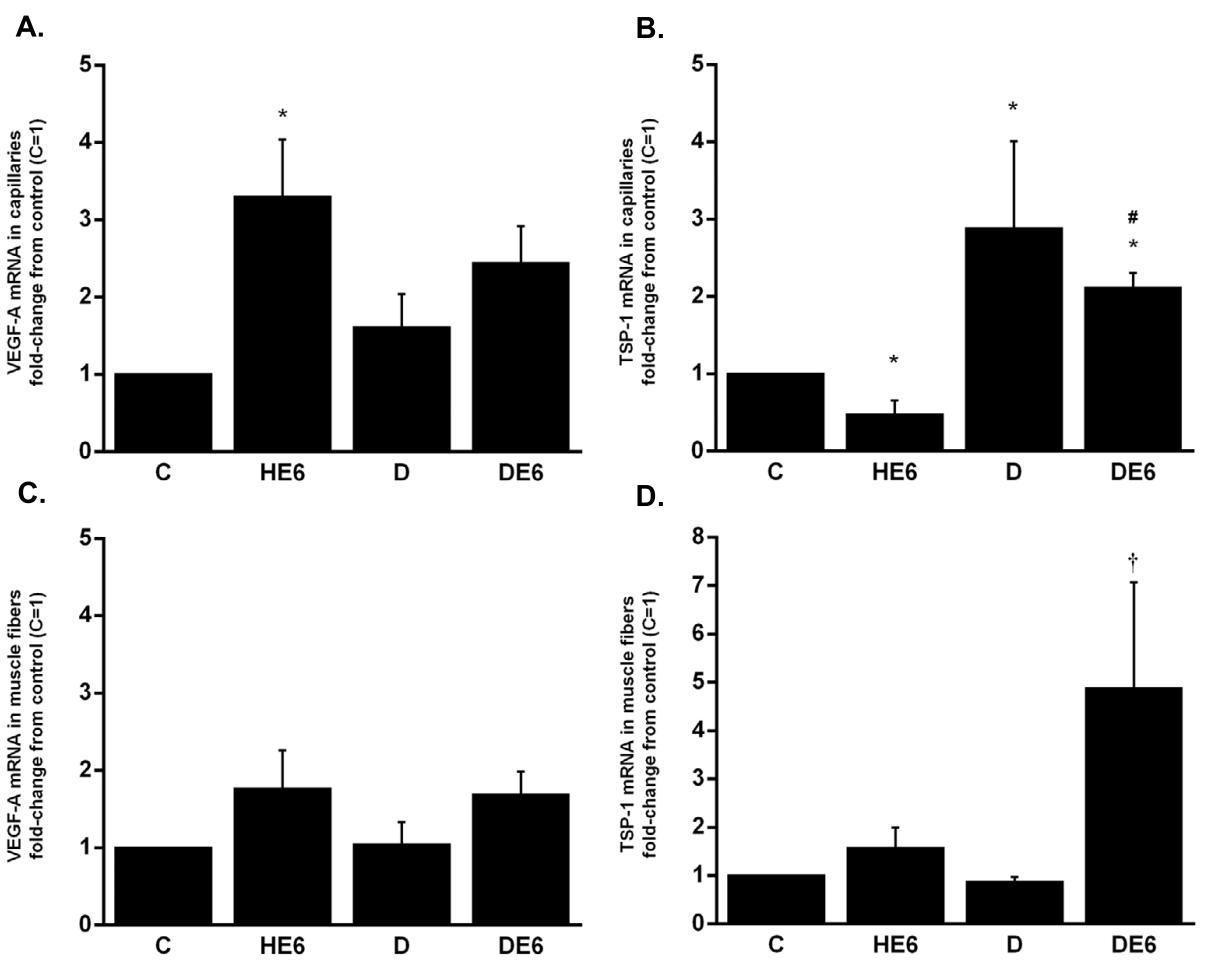
Capillaries and muscle fibers collected from the gastrocnemius muscle with laser capture microdissection were analyzed for the mRNA expression of VEGF-A and TSP-1. Effects of exercise and diabetes in capillaries are shown in images A and B and in muscle fibers in C and D. The results are expressed as a fold-change (mean ± SE) from the healthy control group, which is set to 1. * *P *< 0.05 vs. healthy control, # *P *< 0.05 vs. respective healthy exercised group, † *P *< 0.05 vs. diabetic sedentary.

In the capillaries collected from the gastrocnemius muscle VEGF-A expression increased significantly in HE6 compared to C (Fig. 3a). VEGF-A expression in DE6 did not differ significantly from D, although it was slightly higher in DE6. Capillary expression of TSP-1 decreased in HE6 and increased in D and DE6 compared to healthy controls (Fig. 3b).

In the pure skeletal muscle fibers obtained with LCM, VEGF-A tended to increase in the exercised groups compared to sedentary groups, but these were not statistically significant (Fig. 3C). TSP-1 expression was higher in DE6 than D (*P *< 0.05), and it was also more than 4-fold higher in DE6 compared to the healthy groups, but this was not statistically significant (Fig. 3D).

## Discussion

In the present study we report the effects of a single bout of exercise on the expression of pro- and antiangiogenic factors in diabetic skeletal muscle. The results also provide novel data about exercise- and diabetes-induced changes in the expression of VEGF-A and TSP-1 separately in capillaries and muscle fibers. Our results showed that in the healthy mice the mRNA expression of VEGF-A and its receptor VEGFR-2 were increased, by 25% and 29% respectively, 6 h post exercise in the skeletal muscle homogenates. VEGF-B expression also showed a similar trend (15%, *P *= 0.08). Increases in VEGF-A and VEGFR-2 have been demonstrated earlier in healthy muscles e.g. [16,25-27]. The present results suggest that the expression of VEGF-B may also be affected by exercise; however the increase was not statistically significant. VEGF-B belongs to the same protein family as VEGF-A, and it plays a role in the vascularization of adult and embryonic tissues, particularly in metabolically highly active tissues such as heart and skeletal muscle [28]. However, it has not been studied to the same extent as VEGF-A and its exact role remains yet to be determined. In diabetic mice there was also a trend for increased expression of VEGF-A (17%) and VEGFR-2 (20%) 6 h post exercise, but these changes were not statistically significant. This suggests that angiogenic responses to a single bout of exercise in skeletal muscle are reduced in diabetic compared to healthy mice. The expression of angiogenic growth factors in diabetic muscles may, nevertheless, be upregulated by exercise to some extent. This is supported by the results obtained from our previous exercise training study, where basal mRNA expression of VEGF-A and VEGF-B were increased in trained compared to sedentary diabetic mice [4]. In that study, sedentary diabetic mice exhibited decreased basal expression of VEGF-A and VEGF-B compared to the healthy mice. In the present experiment, there was no difference in the basal expression after 10 days of diabetes, which can be considered as acute diabetes. Contradictory findings exist in the literature about the effects of diabetes or hyperglycemia on the expression of VEGF-A. Several studies have shown decreased expression of VEGF-A in diabetic skeletal muscle or myocardium [9,29-31]. Some studies have reported no change in the expression of VEGF-A due to hyperglycemia [8,32], while others have shown increased expression compared to healthy muscle or myocardium [10,33]. This demonstrates the complexity of the regulation of angiogenic signaling, and how it could be affected by diabetes. The discrepancies between these findings may arise from different models of diabetes used (type 1, type 2 or hyperglycemia), and from the use of different species (human, mouse, rat, rabbit). The severity and duration of diabetes may also have influenced the results. However, despite of the differences between the findings from these studies, it seems that the VEGF signaling pathway is somehow affected in skeletal muscle by diabetes and/or hyperglycemia.

The observed increases in the expression levels of VEGF-A and VEGFR-2 after the exercise bout in the present study were modest compared to several previous studies, where the highest expression (from 2 to 5-fold increase) has been observed within the first hour after the exercise [16,17,27,34-36]. We chose to study time points at 3 h and 6 h post exercise to focus on the recovery period from exercise. At these time points VEGF-A and VEGFR-2 expressions have been shown to remain elevated (see refs above), but for the other proteins studied in the present experiment the data from the recovery period is very limited. We also wanted to study the mRNA expression changes in capillaries when blood flow has returned to basal levels after the exercise bout and angiogenesis processes may proceed. Both animal and human studies have shown that VEGF-A mRNA expression remains elevated up to 4–6 h post exercise, and returns to baseline levels by 8–24 h [16,26,27,37,38]. Different intensity, mode and duration of exercise may also induce variation between the findings of different studies.

The exercise intensity in the present study was chosen so that the diabetic mice were also able to complete the one-hour running bout. Thus, the relative intensity was probably higher for the diabetic mice than for the healthy mice, as their exercise capacity was decreased due to diabetes. However, the changes in the angiogenic gene expression were greater in the healthy mice than in diabetic mice. This further supports the suggestion that angiogenic responses in skeletal muscle are reduced in diabetic mice compared to healthy mice.

The strong correlations found between the proangiogenic factors revealed that mice showing high expression of angiogenic growth factors VEGF-A and VEGF-B, also showed high expression of their receptors, especially VEGFR-2. This suggests that the expression of angiogenic growth factors and their receptors are co-ordinately regulated in response to exercise or elevated blood glucose, and that their expression is controlled by a common upstream regulator. This idea needs to be further studied in the future.

The expression of thrombospondin-1, a known inhibitor of angiogenesis [39], was increased both in the skeletal muscle homogenates and in the capillaries of diabetic mice. Our results concur with the findings by Stenina et al., who found increased TSP-1 expression in the vessel wall of diabetic Zucker rats [40], which is a model for metabolic syndrome. Recently, hexosamine pathway of glucose catabolism was identified to mediate the upregulation of TSP-1 activated by high glucose in human aortic smooth muscle cells [41]. It has been proposed that increased TSP-1 in blood vessels could be a direct response of vascular cells to increased glucose levels and, thus, a link between diabetes and atherosclerotic complications [40,41]. This idea is supported by the present findings, as capillaries from diabetic mice expressed more TSP-1 than capillaries from healthy mice. In addition to atherosclerosis in larger blood vessels, TSP-1 may play a role in capillary rarefaction in skeletal muscles, as has been observed in obese Zucker rats [42] and in type 1 diabetic mice [4]. Interestingly, Olfert et al. recently showed that a single exercise bout increased TSP-1 mRNA in healthy rat skeletal muscle, but that after 3 consecutive days of exercise this response was ablated [43]. In their study the peak expression was detected 1 h after the cessation of exercise, and the increase was no longer statistically significant 2 h post exercise. This concurs with our findings, since we did not observe any changes in TSP-1 expression in healthy mice either 3 h or 6 h after the exercise bout in muscle homogenates. The healthy exercised mice showed even decreased capillary expression of TSP-1 6 h post exercise compared to the sedentary controls (Fig. 3). In addition to increased angiogenic growth factors, the decrease in TSP-1 in endothelial cells may further facilitate angiogenesis processes post exercise. If the decrease in TSP-1 expression occurs only in endothelial cells and not in muscle fibers, it is very difficult to observe from muscle homogenate samples.

The expression of Cyr61 was also increased in diabetic muscles. Cyr61 (also known as CCN1) is an important regulator of angiogenesis, endothelial cell function and ECM modulation [44,45]. It also regulates the activity and production of other angiogenic proteins like VEGF-A [45,46]. Recently, in diabetic retina advanced glycation end products were shown to induce the expression of Cyr61 and connective tissue growth factor (CTGF/CCN2), both of which belong to the same CCN protein family [47]. In the diabetic retina the expression of Cyr61 and CTGF seem to be related to the thickening of capillary basement membrane [47]. If this is also the case in skeletal muscle, it may lead to excess accumulation of extracellular matrix components and endothelial cell death, leading to dysfunctional capillaries and capillary rarefaction. There was a strong positive correlation between blood glucose concentration and the expression of TSP-1 and Cyr61 in the muscle homogenates, indicating that the expression of these genes is related to the level of hyperglycemia. In the present study, exercise did not attenuate the increased expression of either TSP-1 or Cyr61 in diabetic muscle.

On the basis of the present LCM results it seems that the mRNA expression of VEGF-A and TSP-1 is more affected by diabetes and exercise in capillaries than in skeletal muscle fibers. The expression of VEGF-A in capillaries was increased in the healthy exercised mice compared to controls, and thus agreed well with the observations from the muscle homogenates. However, the magnitude of the increase was higher in capillaries. In the muscle fibers extracted with LCM the expression of VEGF-A was increased about 2-fold, but due to the small number of mice in the analysis, it did not reach statistical significance. This increase is similar to what Birot et al. observed in rat single muscle fibers after a bout of endurance exercise (~90%) [48]. Capillary TSP-1 expression was reduced in the healthy exercised group and increased in both diabetic groups compared to healthy controls. These increases in capillary TSP-1 due to diabetes also resemble the changes detected in the muscle homogenates. Taken together, these findings show that diabetes- and exercise-induced effects on angiogenic factors are stronger in capillary endothelial cells than in muscle fibers. This is interesting, since recently Lee and co-workers showed that autocrine VEGF-A signaling in endothelial cells is required for vascular homeostasis, and could not be compensated with paracrine VEGF-A (e.g. from skeletal muscle fibers) [49]. Capillaries in the mouse skeletal muscle are small, and it is possible that cells other than endothelial cells located close to capillaries were also collected in the LCM procedure. However, the majority of the cells in the assay were endothelial cells, and the differences observed in mRNA expression between muscle fibers and capillaries indicate that representative cell populations were collected. This was controlled by setting the laser pulse diameter always smaller than 10 μm and examining the collecting cap microscopically after removing it off the sample.

The reduced capability of diabetic compared to healthy endothelial cells to increase VEGF-A production in response to exercise stimulus may be related to the impaired vascular function and capillary rarefaction in diabetic skeletal muscle. In skeletal muscle and myocardial ischemia studies, diabetic animals exhibit impaired angiogenesis, and this impairment has been linked to several proteins in angiogenesis signaling [5,8-11]. Gene transfer with VEGF-A was able to promote angiogenesis in the myocardium of type 2 diabetic mice [10], but so far angiogenic gene transfer studies in humans have not been very successful [50]. Exercise may positively affect angiogenic signaling by stimulating the whole signaling pathway and thus, be effective in the prevention and treatment of peripheral vascular problems in diseases such as diabetes.

## Conclusion

The present findings showed that angiogenic growth factor response to exercise is attenuated in diabetic compared to healthy mice in skeletal muscle. The present results confirmed our earlier observation that antiangiogenic TSP-1 expression is significantly increased in diabetic muscles. This occurs especially in capillaries, which could be a direct cause to capillary rarefaction seen in diabetic muscles. This study also showed provided evidence that both diabetes and exercise affect the production of angiogenic factors in capillaries and in muscle fibers, the responses being more pronounced in capillaries.

## Competing interests

The authors declare that they have no competing interests.

## Authors' contributions

RK, MS and ML carried out the animal experiment and were involved in the planning of the experiment. RK carried out the qPCR analyses. RK and SJ were involved in the LCM procedure. VV and HK were participated in the design and coordination of the study, and helped to draft the manuscript. All authors read and approved the final manuscript.
